# Groin Puncture to Recanalization Time May Be a Strong Predictor of mTICI 2c/3 over mTICI 2b in Patients with Large Vessel Occlusions Successfully Recanalized with Mechanical Thrombectomy

**DOI:** 10.3390/diagnostics12102557

**Published:** 2022-10-21

**Authors:** Richard Wang, Alperen Aslan, Neda Khalili, Tushar Garg, Apoorva Kotha, Omar Hamam, Meisam Hoseinyazdi, Vivek Yedavalli

**Affiliations:** Department of Radiology and Radiological Sciences, Division of Neuroradiology, Johns Hopkins School of Medicine, Baltimore, MD 21287, USA

**Keywords:** stroke, large vessel occlusions, mechanical thrombectomy, neuro-endovascular, mTICI, recanalization

## Abstract

Mechanical thrombectomy (MT) is an important therapeutic option in the management of acute ischemic stroke (AIS) caused by large vessel occlusions (LVO). While achieving a modified thrombolysis in cerebral infarction (mTICI), grades of 2b, 2c, and 3 are all considered successful recanalization; recent literature suggests that mTICI grades of 2c/3 are associated with superior outcomes than 2b. The aim of this preliminary study is to determine whether any baseline or procedural parameters can predict whether successfully recanalized patients achieve an mTICI grade of 2c/3 over 2b. Consecutive patients from 9/2019 to 10/2021 who were successfully recanalized following MT for confirmed LVO were included in the study. Baseline and procedural data were collected through manual chart review and analyzed to ascertain whether any variables of interest could predict mTICI 2c/3. A total of 47 patients were included in the preliminary study cohort, with 35 (74.5%) achieving an mTICI score of 2c/3 and 12 (25.5%) achieving an mTICI score of 2b. We found that a lower groin puncture to recanalization time was a strong, independent predictor of TICI 2c/3 (*p* = 0.015). These findings emphasize the importance of minimizing procedure time in achieving superior reperfusion but must be corroborated in larger scale studies.

## 1. Introduction

Acute ischemic stroke (AIS) is one of the leading causes of morbidity in the world. Anterior circulation large vessel occlusions (LVOs) cause up to 30% of AIS [1]. In patients presenting with AIS secondary to anterior circulation LVOs, mechanical thrombectomy (MT) is the treatment standard of care if it can be performed within six hours of symptom onset, as well as up to 24 h in selected patients. In these scenarios, MT can lead to smaller infarct volumes and favorable outcomes [2,3,4,5].

It is well established that patients who achieve successful recanalization have favorable clinical outcomes. Historically, patients were considered to be successfully recanalized if they achieved a modified thrombolysis in cerebral infarction (mTICI) grade of 2b, 2c, or 3. However, more recent studies have shown that patients who achieve an mTICI grade of 2c/3—considered excellent recanalization—have better outcomes than those who achieve an mTICI grade of 2b [6,7,8,9]. Is it therefore of growing interest to better understand which factors separate patients who achieve excellent recanalization from those who only achieve mTICI 2b. A few published studies have examined differences in baseline characteristics between mTICI 2b and 3 cohorts [10,11], but no studies to our knowledge has compared mTICI 2b with a combined mTICI 2c and 3 cohort. Given the much superior outcomes that both mTICI 2c and 3 have, further research into this area is greatly needed. Our preliminary study aims to shed light on this topic by determining whether any baseline characteristics or procedural parameters can help predict whether successfully recanalized patients achieve an mTICI grade of 2c/3 over 2b.

## 2. Materials and Methods

### 2.1. Study Population

The study population for this institutional review board (JHU-IRB00269637) approved retrospective study was consecutive patients presenting with AIS with confirmed anterior circulation LVO on CTA who were subsequently treated with MT from September 2019 to August 2021. Recanalization outcomes following MT were scaled with the mTICI grading system based on angiographic appearances. Patients were then sub-grouped into mTICI 2b and combined mTICI 2c/3 for comparative analysis. 

### 2.2. Data Collection

The baseline, clinical, and time parameter data for each patient were collected through manual chart review. The baseline variables collected for each patient included patient demographics, body mass index (BMI), admission National Institutes of Health Stroke Scale (NIHSS), and baseline laboratory values including hemoglobin (Hb), random blood glucose level, blood urea nitrogen (BUN) level, creatinine levels, blood pressure, heart rate, respiratory rate, and blood oxygen level measured with SpO_2_ at admission. The procedural and time parameter data collected for each patient included the door to CT time, the door to groin puncture time, the groin puncture to recanalization time, the number of passes, the type of anesthesia used (general or MAC), and the type of thrombectomy performed (stent retriever, aspiration, or combined). The subtype of stroke in each patient, as defined by the TOAST criteria, was additionally collected. In patients who received a thrombolytic agent, the door to needle time was collected. ASPECTS scores on baseline non-contrast CT and site of occlusion on CT angiography (CTA) were assessed by a board-certified neuroradiologist (V.S.Y., 6 years of experience).

### 2.3. Study Outcomes

The primary outcome measure was the likelihood of excellent recanalization, defined as mTICI grade 2c/3.

### 2.4. Statistical Analysis

The data were managed and analyzed using IBM SPSS statistics (Statistical Package for Social Sciences) software version 26, IBM Corp., Chicago, IL, USA, 2013 and Microsoft Office Excel 2007.

Quantitative data were described using means ± SD (standard deviation), then compared using independent t-tests. Qualitative data were described using numbers and percentages, then compared using Chi-squared and Fisher’s Exact tests. Normal distribution of data was evaluated using the Shapiro-Wilk test. Univariate analysis was initially applied to examine each of the baseline variables independently. Logistic regression (backward stepwise method) was performed to determine the independent factors for predicting mTICI grade 2c/3 after entering variables from the univariate analysis with *p*-value < 0.1, as well as age and sex. *p*-values of < 0.05 were considered significant.

## 3. Results

A total of 47 patients were included in the study cohort. Among these cases, 35 (74.5%) achieved an mTICI score of 2c/3, and 12 (25.5%) achieved an mTICI score of 2b.

Table 1 shows the baseline characteristics of the patient cohort with comparison between patients who achieved mTICI grade 2c/3 and 2b. The mean ± SD age of the studied cases was 67.8 ± 14.4 years.

Table 2 shows the differences in time parameter and related intervention data between the mTICI 2c/3 and mTICI 2b cohorts. By univariate analysis, patients who achieved mTICI 2c/3 recanalization had a significantly lower groin puncture to recanalization time than those who achieved mTICI 2b (32.4 ± 20.3 vs. 51.4 ± 28.5 min, *p* = 0.02). In addition, type of thrombectomy significantly differed between patients in the mTICI 2c/3 and mTICI 2b groups (*p* = 0.05).

After adjusting for potential confounders, groin puncture to recanalization time and IV tPA administration were significantly associated with mTICI 2c/3 (*p* = 0.015 and *p* = 0.037, respectively) (Table 3). Based on the logistic regression model, every one-minute increase in groin puncture to recanalization time was associated with a 4.2% decrease in the likelihood of achieving mTICI 2c/3. Administration of IV tPA was also associated with a decrease in the likelihood of achieving mTICI 2c/3.

## 4. Discussion

In this preliminary, single-center, retrospective study, a lower groin puncture to recanalization time was a strong, independent predictor of excellent recanalization.

In patients presenting with AIS secondary to LVOs, timely MT is the mainstay treatment in achieving recanalization. While it is recognized that mTICI 2c/3 patients have improved outcomes over mTICI 2b patients [7,8,9], little is known about what factors drive this difference in recanalization. Our study aimed to shed light on this topic by examining whether any baseline characteristics or procedural parameters were predictive of patients achieving mTICI 2c/3 over 2b following endovascular treatment.

Importantly, we found that patients who achieved excellent recanalization had a lower groin puncture to recanalization time than those in the mTICI 2b group. The mTICI 2c/3 group had an average groin puncture to recanalization time of 32.4 ± 20.3 min and the mTICI 2b group had an average groin puncture to recanalization time of 51.4 ± 28.5 min, a nearly twenty-minute average difference. In addition, our logistic regression model showed that every one-minute increase in groin puncture to recanalization time reduced the likelihood of excellent recanalization by 4.2% (*p* = 0.015). While a previous study showed that longer MT procedure times are associated with mTICI 3 over 2b [10], this is the first time that this relationship has been shown to extend to a combined mTICI 2c and 3 cohort as well. Interestingly, no other time parameters, including door to CT, door to groin puncture, or door to needle, were either associated with or predictive of excellent recanalization. Additionally, there were no significant differences in the mechanism of stroke or the type of thrombectomy performed between the mTICI 2c/3 and 2b cohorts, both of which may influence the operative time. This suggests that in patients with successful reperfusion, the procedure time itself is the primary driver of how much reperfusion is achieved. One possible explanation for this finding is that longer surgery times may increase the risk of neurological stress through a variety of mechanisms, such as prolonged anesthesia exposure or altered cerebral perfusion, and thus lead to worse reperfusion [12,13]. Indeed, surgery has been shown to induce a stress response that results in derangements of various metabolic and inflammatory processes [14]. For example, it has been shown that surgery increases the risk of neuroinflammation through both widespread microglia activation and facilitation of entry of inflammatory cytokines by damaging the blood brain barrier [15,16,17]; this neuroinflammation may affect the extent of reperfusion achieved. Shorter procedure times may also simply reflect greater surgical skill of the operator, which would consequently result in more procedural success. Ultimately, shortening the operative time is a key target for optimization to improve chances of excellent recanalization after MT.

Of note, our logistic regression model also showed that IV tPA administration was a negative predictor of excellent recanalization (*p* = 0.37). This finding is unintuitive, and it most likely represents the small sample size of patients in the study overall, as well as the discrepancy in the proportion of patients who received IV tPA administration between the mTICI 2b group and the mTICI 2c/3 group (58.3% and 25.7% of the cohort, respectively). We will investigate this relationship further in a future, larger scale study.

Our study design has several limitations to acknowledge. First, this study only included patients from a single center, which may have led to sampling bias. We attempted to minimize this by including consecutive patients. Secondly, the study only included patients who received MT following an acute LVO of the intracranial ICA as well as the M1 and proximal M2 segments of the MCA. Thirdly, due to the strictness of our selection criteria, our study only included 47 patients, which may have limited the power of our study. For that reason, our results should be interpreted with caution and further corroborated with larger scale studies.

In conclusion, we found that a lower groin puncture to recanalization time was a strong, independent predictor of excellent recanalization. While timeliness is necessary for all stroke interventions, our findings emphasize the particular importance of minimizing MT procedural time in achieving superior reperfusion. Our preliminary findings may have implications for the management of AIS secondary to LVO treated with MT with the objective of achieving excellent recanalization. However, larger scale studies must be performed to further assess the strength of these findings.

## Figures and Tables

**Table 1:** Descriptive statistics among the studied cases and comparison according to mTICI grade.

Variables			All Cases (*n* = 47)	mTICI Grade		*p*-Value
				2c/3 (*n* = 35)	2b (*n* = 12)	
Age (years)			67.8 ± 14.4	69.3 ± 15.0	63.3 ± 12.2	0.213
Sex (*n*, %)	Male		25 (53.2)	20 (57.1)	5 (41.7)	0.354
	Female		22 (46.8)	15 (42.9)	7 (58.3)	
Race (*n*, %)	White/Caucasian		27 (57.4)	21 (60.0)	6 (50.0)	0.649
	African		19 (40.4)	13 (37.1)	6 (50.0)	
	Asian		1 (2.1)	1 (2.9)	0 (0.0)	
BMI (kg/m^2^)			28.0 ± 8.4	27.5 ± 7.9	29.5 ± 10.1	0.476
BMI grade (*n*, %)		<30.0	32 (68.1)	25 (71.4)	7 (58.3)	0.481
		≥30.0	15 (31.9)	10 (28.6)	5 (41.7)	
Hemoglobin (gm/dL)			12.6 ± 1.9	12.4 ± 2.1	13.3 ± 1.3	0.159
Glucose (mg/dL)			135.0 ± 68.2	133.5 ± 65.5	139.6 ± 78.6	0.792
BUN/creatinine ratio			17.7 ± 7.2	18.3 ± 7.6	16.1 ± 5.7	0.372
SBP (mmHg)			149.3 ± 23.7	147.9 ± 21.3	153.2 ± 30.5	0.517
DBP (mmHg)			84.4 ± 19.3	82.7 ± 19.8	89.5 ± 17.6	0.297
HR (beats/min)			81.2 ± 18.6	82.9 ± 18.8	76.5 ± 17.7	0.311
RR (cycles/min)			17.6 ± 3.7	17.2 ± 3.9	18.8 ± 3.1	0.200
SPO_2_ (%)			98.0 ± 2.3	97.7 ± 2.4	98.7 ± 1.9	0.222
Admission NIHSS			15.5 ± 7.0	16.0 ± 6.2	13.9 ± 9.0	0.373
ASPECTS			8.7 ± 2.0	8.9 ± 1.9	8.3 ± 2.2	0.314
Subtype of ischemic stroke per TOAST criteria (*n*, %)		Large artery atherosclerosis	7 (14.9)	3 (8.6)	4 (33.3)	0.136
		Cardioembolic	30 (63.8)	25 (71.4)	5 (41.7)	
		Small-vessel occlusion	0 (0)	0 (0)	0 (0)	
		Stroke of other determined etiology	1 (2.1)	1 (2.9)	0 (0)	
		Stroke of undetermined etiology	9 (19.1)	6 (17.1)	3 (25.0)	
Site (*n*, %)		Right	20 (42.6)	17 (48.6)	3 (25.0)	0.154
		Left	27 (57.4)	18 (51.4)	9 (75.0)	
48 h post-MT HT, (*n*, %)			16 (34.0)	11 (32.4)	5 (41.7)	0.726

BMI, body mass index; BUN, blood urea nitrogen; SBP, systolic blood pressure; DBP, diastolic blood pressure; HR, heart rate; RR, respiratory rate; HT, hemorrhagic transformation.

**Table 2:** Comparison of intervention and time parameter data between the mTICI 2b and mTICI 2c/3 cohorts.

Variables		All Cases (*n* = 47)	mTICI Grade		*p*-Value
			2c/3 (*n* = 35)	2b (*n* = 12)	
Door to CT (mins)		32.3 ± 30.9	35.5 ± 33.7	22.0 ± 16.6	0.209
Door to groin puncture (mins)		167.0 ± 112.7	158.8 ± 72.7	190.3 ± 188.0	0.412
Groin puncture to recanalization (mins)		37.6 ± 24.0	32.4 ± 20.3	51.4 ± 28.5	0.017 *
Number of passes		1.4 ± 1.1	1.4 ± 1.0	1.6 ± 1.2	0.588
Type of anesthesia (*n*, %)	General	40 (85.1)	31 (88.6)	9 (75.0)	0.350
	MAC	7 (14.9)	4 (11.4)	3 (25.0)	
Type of Thrombectomy (*n*, %)	Direct Aspiration	30 (63.8)	26 (74.3)	4 (33.3)	0.053 *
	Stent Retriever	1 (2.1)	1 (2.9)	0 (0)	
	Combined	13 (27.7)	7 (20.0)	6 (50.0)	
IV tPA administered (*n*, %)		16 (34.0)	9 (25.7)	7 (58.3)	0.075
Door to needle time (mins)		66.9 ± 43.4	80.6 ± 52.5	49.3 ± 19.5	0.159

CT, computed tomography; IV tPA, intravenous tissue-type plasminogen activator. * Significant (*p* < 0.05).

**Table 3:** Multivariate logistic regression of studied variables in predicting mTICI 2c/3 in all patients.

Variable	OR	95% C.I.		*p*-Value
		Lower	Upper	
Time from groin puncture to recanalization	0.0958	0.926	0.992	0.015 *
IV tPA administered	0.132	0.020	1.072	0.037 *
Constant	0.872	N/A	N/A	0.945

OR, odds ratio; C.I., confidence interval. * Significant (*p* < 0.05).

## Data Availability

Not applicable.
